# Validation of the Telephone MoCA in iRBD and Parkinson's Disease with Conversion to the Standard MoCA

**DOI:** 10.1002/mdc3.70905

**Published:** 2026-10-30

**Authors:** Ludo van Hillegondsberg, Tanja Zerenner, Michael Lawton, Karolien Groenewald, Jamil Razzaque, Jessica Welch, Yoav Ben‐Shlomo, Michele Hu

**Affiliations:** ^1^ Nuffield Department of Clinical Neurosciences University of Oxford Oxford UK; ^2^ Population Health Sciences University of Bristol Bristol UK

**Keywords:** isolated REM sleep behavior disorder, Montreal cognitive assessment, Parkinson's disease, telephone cognitive assessment

## Abstract

**Background:**

Telephone‐based cognitive assessment may improve follow‐up when face‐to‐face testing is impractical. The Telephone Montreal Cognitive Assessment (T‐MoCA) omits visually dependent items from the standard MoCA, but its validity in isolated REM sleep behavior disorder (iRBD) and Parkinson's disease (PD), remains uncertain.

**Objectives:**

To assess agreement between the T‐MoCA and face‐to‐face MoCA‐22 in iRBD and PD, and derive and externally validate a MoCA‐22 to MoCA‐30 conversion.

**Methods:**

In the Oxford Parkinson's Disease Centre (OPDC) Discovery Cohort, 137 participants (38 controls, 47 iRBD, 52 PD) completed T‐MoCA and MoCA‐22 assessments. Agreement was evaluated using Lin's concordance correlation coefficient, Bland–Altman analysis, and equivalence testing with ±1‐point bounds. Equipercentile equating with log‐linear smoothing was used to derive a MoCA‐22 to MoCA‐30 conversion using Discovery data (3561 tests), with validation in Tracking Parkinson's (5767 tests) and Parkinson's Progression Markers Initiative (5047 tests).

**Results:**

T‐MoCA and face‐to‐face MoCA‐22 scores were statistically equivalent, with minimal bias; 83.3% of scores differed by ≤1 point. However, concordance between test formats was moderate (Lin's CCC = 0.58). Across 14,375 MoCA assessments, equipercentile equating outperformed proportional scaling in development and validation cohorts. Propagating T‐MoCA–MoCA‐22 variability resulted in wider prediction intervals for T‐MoCA‐derived MoCA‐30 estimates, indicating individual‐level uncertainty, with intervals estimable only for T‐MoCA scores ≥17 due to sparse data.

**Conclusions:**

At group level, T‐MoCA is a valid remote measure of non‐visual MoCA domains in iRBD and PD. The equating function supports cohort‐level harmonization of MoCA‐22 and MoCA‐30 scores, while individual T‐MoCA‐derived MoCA‐30 estimates should be interpreted with caution.

Identifying cognitive impairment is of critical importance across a wide range of clinical settings, where early detection informs diagnosis, prognosis, and management.^1^ The Montreal Cognitive Assessment (MoCA) is one of the most widely used and well‐validated screening tools for cognitive impairment, demonstrating strong sensitivity for detecting dementia.^2^, ^3^ However, the standard MoCA requires face‐to‐face administration, limiting its feasibility when in‐person assessment is impractical. Barriers such as reduced mobility, geographic distance, intercurrent illness, or public health restrictions have underscored the need for reliable and validated remote cognitive assessment tools. In response, a variety of digital and online cognitive screening instruments have been developed in recent years.^4^ While these tools offer innovative approaches to remote assessment, they often require a degree of computer literacy, access to appropriate technology, and sufficient preserved cognitive function to engage meaningfully with the testing platform.^5^ These requirements may limit their applicability in older or cognitively vulnerable populations and simpler remote approaches, such as brief telephone‐based cognitive assessments, offer clear practical advantages in these settings. One such instrument is the Telephone Montreal Cognitive Assessment (T‐MoCA), a remote adaptation of the standard MoCA designed for administration via telephone. The T‐MoCA retains the verbally administered components of the original test while omitting the visuospatial/executive and naming sections, thereby only having a maximum available score of 22.^6^, ^7^ Previous studies have supported the validity and clinical utility of the T‐MoCA as cognitive screening tool across several populations, including community‐dwelling older adults and individuals with transient ischaemic attack and stroke.^8^, ^9^, ^10^, ^11^ A related methodological challenge is how to compare scores from the T‐MoCA with scores from the standard 30‐point MoCA. Katz et al. addressed this issue in a diverse community‐based cohort of older adults, demonstrating statistical equivalence between the T‐MoCA and the face‐to‐face MoCA‐22, and proposing two approaches for estimating standard MoCA‐30 scores from T‐MoCA scores: equipercentile equating with log‐linear smoothing, and education‐adjusted Poisson regression.^8^ Their work provides an important framework for harmonizing remote and in‐person MoCA data, but conversion functions may not generalize across clinical populations, particularly where cognitive profiles differ from those of community‐dwelling older adults. Further validation in disease‐specific cohorts is therefore necessary. Despite this growing evidence base, the application of the T‐MoCA in Parkinson's disease remains relatively underexplored. Preliminary work suggests that the T‐MoCA measures may help identify mild cognitive impairment in Parkinson's disease, but the single available study requires further validation in larger, well‐characterized cohorts.^12^ Cognitive impairment is a common non‐motor feature of PD, and detecting it timeously has important care implications.^13^, ^14^ In isolated REM‐sleep behavior disorder (iRBD), a widely recognized form of prodromal PD, no studies exist validating the T‐MoCA.^15^, ^16^, ^17^

This study aimed to evaluate the validity of the T‐MoCA in participants with iRBD and Parkinson's disease enrolled in the Oxford Parkinson's Disease Centre (OPDC) Discovery Cohort. Specifically, we assessed agreement between the T‐MoCA and a modified face‐to‐face MoCA comprising the same 22 non‐visual items (MoCA‐22) to allow direct comparability between administration formats. We also aimed to derive and externally validate an equating function to convert MoCA‐22 scores to standard MoCA scores using three large PD cohorts.

## Methods

### Study Population

This study utilized data from three longitudinal Parkinson's disease (PD) cohorts: the OPDC Discovery cohort, Tracking Parkinson's, and the Parkinson's Progression Markers Initiative (PPMI). OPDC Discovery recruited recently diagnosed patients with PD from 11 hospitals across the Thames Valley between September 2010 and January 2016, while Tracking Parkinson's recruited recently diagnosed patients across the UK between February 2012 and May 2014. In both cohorts, PD was diagnosed by an NHS neurology consultant and confirmed at enrolment using the UK Brain Bank Criteria.^18^ Participants were reviewed every 12–18 months, and those who developed atypical parkinsonian syndromes were withdrawn. In OPDC Discovery, individuals with iRBD were referred from regional sleep clinics and had their diagnosis confirmed via polysomnography before enrolment, in accordance with the International Classification of Sleep Disorders criteria.^19^ Both studies have been described previously.^20^, ^21^ PPMI is an ongoing international observational study comprising healthy controls, individuals with de novo PD diagnosed according to UK Brain Bank Criteria, prodromal PD participants, and non‐manifesting carriers of *LRRK2* and *GBA* variants. Participants undergo clinical, imaging, and molecular assessments at 6‐ or 12‐month intervals. Study protocols are available at https://www.ppmi-info.org/study-design. Data were obtained on March 13, 2026 from the PPMI database (www.ppmi-info.org/access-dataspecimens/download-data), RRID:SCR_006431.

For the T‐MoCA sub‐study, OPDC Discovery participants were recruited on a rolling basis during scheduled study visits. Exclusion was limited to profound visual or hearing impairment precluding assessment. PD participants were tested while on treatment. For derivation and validation of the equating formula, all PD cases considered sporadic at enrolment were included.

### Cognitive Tests

Participants completed the T‐MoCA, comprising all standard MoCA items except the visually dependent visuospatial/executive and naming components, and the standard 30‐point MoCA (MoCA‐30). The T‐MoCA was performed by telephone, with the participant and the test administrator located in separate clinic rooms, out of earshot. The T‐MoCA was administered by telephone with participant and administrator in separate clinic rooms. T‐MoCA and face‐to‐face MoCA‐22 were administered at opposite ends of the study visit using different test versions to minimize practice effects. Within diagnostic groups, consecutively recruited participants were allocated to alternating test‐order groups. Routine motor and cognitive assessments were performed between the two MoCA administrations. Following completion of the MoCA‐30, a 22‐point subscore (MoCA‐22) was derived by excluding the visuospatial/executive and naming items omitted for the T‐MoCA, thereby permitting direct comparison of performance on the same non‐visual items across administration formats.

### Statistical Analysis

Demographic characteristics of the population included in the T‐MoCA sub‐study were analyzed to assess baseline differences. Distributions of continuous variables across groups were compared using the Kruskal‐Wallis test and binary variables were evaluated with Pearson's Chi‐squared test. Paired T‐MoCA and MoCA‐22 scores were compared using the Wilcoxon signed‐rank test. Further statistical analyses were conducted to evaluate concordance, agreement and equivalence between face‐to‐face (F2F) and telephone administration of the MoCA‐22. Correlation was assessed using the Spearman's rank correlation coefficient, and concordance was quantified using Lin's Concordance Correlation Coefficient (CCC), which assesses both precision and accuracy relative to the line of identity. Agreement was further examined using Bland–Altman plots, with calculation of the mean bias and 95% limits of agreement (LoA), including confidence intervals, to assess systematic differences. Formal equivalence testing was performed using the Two One‐Sided Tests (TOST) procedure, with prespecified equivalence bounds of ±1 MoCA point. These bounds were defined a priori based on clinically negligible differences and expected measurement error.^22^, ^23^ The potential effects of test format and order were tested using a linear mixed effects model, with subject ID set as the random intercept.

Equipercentile equating with log‐linear smoothing was used to derive a MoCA‐22‐to‐MoCA‐30 conversion in OPDC Discovery. Model performance at the individual‐level was evaluated by comparing equated and observed MoCA‐30 scores using root mean squared error (RMSE) and mean absolute error (MAE). Both metrics quantify the average discrepancy between observed and predicted scores, with lower values indicating better agreement. Distributional agreement between observed and equated scores was assessed using the Kolmogorov–Smirnov (KS) and Cramér–von Mises (CvM) statistics, which capture maximum and overall differences between the empirical score distributions; and the Wasserstein distance (W_2_), which measures the average difference between distributions on the MoCA scale. Again, smaller values are better. Performance was compared with proportional scaling (MoCA‐22 × 30/22) and externally validated in PPMI and Tracking Parkinson's using the same metrics.

To quantify individual‐level uncertainty, 95% prediction intervals were estimated for MoCA‐22‐derived MoCA‐30 scores and for MoCA‐30 estimates derived from the T‐MoCA. For each F2F MoCA‐22 score, the 2.5th and 97.5th percentiles of the corresponding observed MoCA‐30 distribution in the OPDC Discovery cohort defined the empirical prediction interval. For T‐MoCA, uncertainty across both stages was propagated using 10,000 Monte Carlo iterations, sampling an observed T‐MoCA–F2F MoCA‐22 difference to generate a plausible F2F MoCA‐22 score, followed by a corresponding observed MoCA‐30 score from the OPDC Discovery cohort. The 2.5th and 97.5th percentiles of this distribution defined the prediction interval. The pooled T‐MoCA–F2F MoCA‐22 error distribution was predominantly informed by higher T‐MoCA scores, therefore intervals were reported only for scores with >10 observations. When assessing the clinical impact of score conversion, observed and equated MoCA‐30 scores were categorized as dementia (≤20), mild cognitive impairment (MCI; 21–25), or normal cognition (≥26). Agreement between observed and equated classifications was summarized as the proportion of assessments with concordant classifications, Cohen's *κ*, and the frequency and direction of classification changes. Linear mixed‐effects models were used to examine the effects of relevant participant and study variables on the observed results, while accounting for repeated within‐participant measurements. To assess the influence of repeated observations, participant‐level bootstrap analyses randomly selected one assessment per participant and repeated equating and validation. Statistical analyses were performed using R version 4.3.1, with the TOSTER (v0.8.3), BlandAltmanLeh (v0.3.1), epiR (v2.0.78), equate (v2.0.8), lme4 (v1.1–35.5), and psych (v2.4.12) packages.

## Results

The T‐MoCA sub‐study included 137 participants (38 healthy controls, 47 iRBD, 52 PD). Groups were similar in age, but the iRBD group had a significantly higher proportion of males. MoCA scores tended to be lower in PD than controls, while test order was comparable across groups and time between tests differed significantly. Ten iRBD participants subsequently phenoconverted: seven to PD (mean 1.7 years) and three to dementia with Lewy bodies (mean 1.0 year; Table 1). The order × format interaction showed that when the T‐MoCA was administered first, T‐MoCA scores tended to be higher, and when the face‐to‐face MoCA‐22 was administered first, face‐to‐face scores tended to be higher (Supplementary Table 1). This pattern does not suggest a consistent practice effect but rather is compatible with a possible fatigue effect that lowered scores on the second test. Given the imbalance in sex distribution, we additionally examined the association between sex and cognitive performance. Across the full sample, males had significantly lower T‐MoCA scores than females (*β* = −0.99, *p* = 0.016), although there was no evidence that this sex difference varied by diagnostic group. There was no significant overall sex difference in MoCA‐22 scores, nor by diagnostic group (Supplementary Table 2). However, it is worth noting that the iRBD sex comparison is based on three females versus 44 males, and so a non‐significant result in such an imbalanced group should not be interpreted as demonstrating the absence of a sex effect. Notwithstanding this, there was no overall difference between T‐MoCA and MoCA‐22 scores (*p* = 0.80; median absolute difference = 1), nor within diagnostic groups (HC: *p* = 0.94; iRBD: *p* = 0.17; PD: *p* = 0.67). The TOST analyses further indicated that T‐MoCA and MoCA‐22 scores were statistically equivalent within the pre‐specified ±1 MoCA point equivalence bounds in the full sample (*p* < 0.001) and across all diagnostic groups (HC: *p* = 0.002; iRBD: *p* = 0.044; PD: *p* = 0.004).

**TABLE 1:** Telephone MoCA sub‐study demographics (OPDC discovery cohort)

	Healthy control (n = 38)	iRBD (n = 47)^a^	Parkinson's disease (n = 52)	*p*‐value
Age	62 (53, 66)	64 (58, 69)	62 (54, 68)	0.3
Sex (no. male)	18 (47%)	44 (94%)	27 (52%)	<0.001
Disease duration (yrs)	‐	2 (1, 4)	11 (9,13)	‐
Education (yrs)	16 (14, 19)	16 (12, 17)	15 (12,17)	
UPDRS III	1 (0, 3)	10 (5, 15)	36 (24,42)	<0.001
Hoehn & Yahr Stage				
1	‐	‐	7 (14%)	
2	‐	‐	30 (60%)	
3	‐	‐	10 (20%)	
4	‐	‐	3 (6%)	
LEDD (mg/day)	‐	‐	700 (500,1040)	
MoCA score	28 (26, 29)	27 (24, 29)	26 (25, 28)	0.05
MoCA‐22 score	20 (18, 21)	19 (17, 21)	19 (18, 21)	0.2
T‐MoCA score	20 (18, 21)	19 (18, 21)	19 (17, 21)	0.3
Test order				0.9
MoCA‐22 first	20 (53%)	26 (55%)	26 (50%)	
T‐MoCA first	18 (47%)	21 (45%)	26 (50%)	
Time between tests (min)	80 (65, 89)	65 (59, 109)	96 (83, 117)	0.01

*Note*: Continuous values expressed as median (Q1, Q3). Disease duration measured from date of diagnosis. Missingness: 3 PD cases did not have up‐to‐date motor data, and 6 LEDD data.

Abbreviations: MoCA, Montreal Cognitive Assessment; T‐MoCA, Telephone Montreal Cognitive Assessment; UPDRS, Unified Parkinson's Disease Rating Scale.

^a^ This arm includes 10 phenoconverters. 7 converted to PD (mean time from testing to conversion: 1.7 years) and 3 to Dementia with Lewy Bodies (mean time from testing to conversion: 1.0 year).

Spearman rank's correlation coefficient and Lin's CCC indicated moderate overall correlation and concordance between MoCA‐22 and T‐MoCA scores (*ρ* = 0.50; CCC = 0.58, 95% CI [0.46, 0.68]). Correlation and concordance were highest in the iRBD group (*ρ* = 0.58; CCC = 0.67, 95% CI [0.49, 0.80]) followed by PD (*ρ* = 0.44; CCC = 0.51, 95% CI [0.27, 0.68]) and HC (*ρ* = 0.49; CCC = 0.47, 95% CI [0.18, 0.68]). Bland–Altman analysis demonstrated generally good agreement between T‐MoCA and MoCA‐22 scores across the full sample and diagnostic groups, with 83.3% of test pairs differing by no more than one point and 94.1% falling within the limits of agreement. There was little evidence of systematic bias, as the mean differences were close to zero in all groups (Fig. 1).

**Figure 1:**
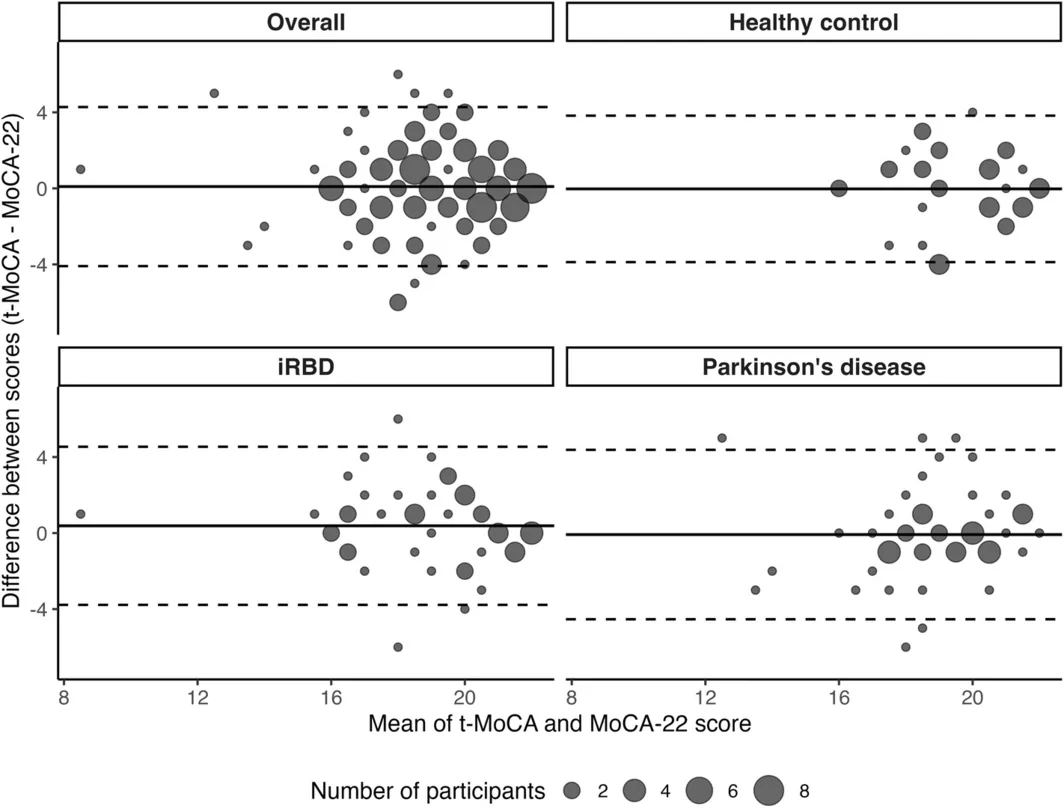
Bland–Altman plots of T‐MoCA and face‐to‐face MoCA‐22 scores.

### Derivation of the Equating Function

To develop and validate a method to convert MoCA‐22 scores to standard MoCA scores, we used longitudinal MoCA data from three independent PD cohorts: the OPDC Discovery cohort, the Parkinson's Progression Markers Initiative (PPMI) cohort, and the Tracking Parkinson's cohort. In total, 14,375 MoCA assessments were available from 4045 PD participants across the three datasets. Median years of education was significantly lower in the OPDC Discovery cohort compared to the PPMI (13 vs 16; p < 0.001). Consistent with this, the proportion of participants with ≤12 years of education was highest in OPDC Discovery (40%), followed by Tracking Parkinson's (32%) and PPMI (12%; *p* < 0.001). Median MoCA scores also differed significantly between cohorts (p < 0.001), with tests in the OPDC Discovery and Tracking Parkinson's cohorts capturing lower scores overall compared with those in the PPMI cohort (Table 2 and Supplementary Fig. 1). Given the differences in educational attainment between cohorts, we examined whether education modified the relationship between MoCA‐22 and MoCA‐30. No significant interaction was observed in the OPDC Discovery cohort and small interactions were detected in the Tracking Parkinson's and PPMI cohorts, collectively providing no consistent evidence that educational attainment materially influenced the MoCA‐22–MoCA‐30 relationship (Supplementary Table 3). Across all cohorts, points were predominantly lost on non‐visual rather than visual items (Supplementary Fig. 2 and Supplementary Table 4). Utilizing the OPDC Discovery MoCA data, equipercentile equating with log‐linear smoothing (degree = 3) was used to derive a conversion from MoCA‐22 to MoCA‐30 scores. The resulting conversion table mapped each MoCA‐22 score to an estimated MoCA‐30 score (Table 3). Equated and observed MoCA‐30 scores showed a Spearman's *ρ* of 0.95, Lin's CCC of 0.95, RMSE of 1.16 and MAE of 0.85. Distributional fit statistics were as follows: Kolmogorov–Smirnov statistic 0.060, Cramér–von Mises statistic 0.012, and Wasserstein distance 0.59. For comparison, proportional scaling using the formula MoCA‐22 × (30/22) produced a Spearman's *ρ* of 0.95, Lin's CCC of 0.95, RMSE of 1.30 and an MAE of 0.97. The corresponding distributional fit statistics were 0.094 for the Kolmogorov–Smirnov statistic, 0.027 for the Cramér–von Mises statistic, and 0.77 for the Wasserstein distance (Table 4). The relationship between observed MoCA‐22 and MoCA‐30 scores and the derived equating function is illustrated in Figure 2 and agreement between observed and equated MoCA‐30 scores is shown in Figure 3. Predicted scores were generally close to observed scores across the represented range. However, MoCA‐22 scores ≤5 were very sparsely represented in the derivation cohort. Estimates at these score levels therefore rely predominantly on extrapolation of the smoothed equating function and should be interpreted with caution. As a sensitivity analysis, we both derived the MoCA‐22‐to‐MoCA‐30 equating function using longitudinal iRBD data and applied the PD‐derived equating function to the iRBD arm of the OPDC Discovery cohort. Both cases demonstrated broadly comparable equating accuracy to that observed in PD, and although the equating residual differed slightly between groups in a mixed‐effects model (*β* = 0.19, p < 0.001), the magnitude of this difference was small (less than one‐fifth of a MoCA point), supporting the performance of the PD‐derived conversion in iRBD (Supplementary Tables 5–7). Higher age was associated with a slightly greater equating residual (*β* = 0.013 points/year, *p* < 0.001), whereas sex (*β* = 0.100, *p* = 0.063) and years of education (*β* = −0.011, *p* = 0.142) showed no significant association (Supplementary Table 8). After adjustment for age, sex, education, and repeated within‐participant assessments, equating residual was not associated with disease duration (*β* = 0.005 MoCA points/year, SE = 0.006, *p* = 0.443). Although estimates at longer disease durations may have been influenced by greater attrition, no clear trend of increasing conversion bias with disease duration was noted. Beyond 10 years, the equated score tended to underestimate the observed MoCA‐30 score, consistent with proportionally greater loss on non‐visual items among those remaining in follow‐up (Supplementary Fig. 3). To quantify individual‐level uncertainty, 95% prediction intervals were generated for MoCA‐22‐derived MoCA‐30 scores and, separately, for T‐MoCA‐derived estimates by propagating observed T‐MoCA–MoCA‐22 variability through the conversion. T‐MoCA‐derived intervals were substantially wider (Table 3). Intervals are reported only for scores with >10 observations to avoid unstable estimates from sparse data.

**TABLE 2:** Longitudinal MoCA data of PD participants from the three cohorts

	OPDC Discovery	Tracking PD	PPMI	p‐value
No. of PD participants	1003	1907	1135	
Age (years)	68 (62, 75)	68 (63, 74)	64 (57, 70)	<0.001
Sex (n, % male)	646 (64%)	1242 (65%)	736 (65%)	>0.9
Education (yrs)	13 (11, 16)	n.a	16 (14, 18)	<0.001
≤ 12 years of education	397 (40%)	578 (30%)	136 (12%)	<0.001
No. of tests	3561	5767	5047	
MoCA‐30 score	25 (23, 28)	26 (24, 28)	28 (26, 29)	<0.001
MoCA‐22 score	18 (16, 20)	19 (17, 21)	20 (18, 21)	<0.001
MoCA‐30/MoCa‐22 correlation	0.95	0.96	0.93	

*Note*: MoCA and MoCA‐22 scores are reported as median (Q1, Q3), calculated using all available assessments conducted in PD participants. n.a = not available. Missingness: Education data missing for 9 Discovery, 94 Tracking PD and 3 PPMI participants. Correlation assessed using Spearman's rank correlation coefficient (*ρ*).

**TABLE 3:** Equipercentile conversion table derived from the OPDC Discovery cohort with accompanying prediction intervals

Observed MoCA‐22	Estimated MoCA‐30	MoCA‐22 Percentile	MoCA‐30 Percentile	MoCA‐22‐derived MoCA‐30 95% prediction interval^a^	T‐MoCA‐derived MoCA‐30 95% prediction interval^b^
0	0	0	0		
1	1	0	0		
2	2	0.1	0		
3	3	0.1	0		
4	5	0.1	0.1		
5	7	0.2	0.1		
6	9	0.4	0.3		
7	11	0.5	0.6		
8	12	0.8	0.7	10–14	
9	13	1.3	1.1	12–16	
10	15	1.9	2.2	12–18	
11	16	3.3	3.3	14–19	
12	17	5.2	4.8	14–20	
13	19	8.7	9.5	16–21	
14	20	13.3	12.8	17–22	
15	21	19.7	17.8	18–23	
16	22	28.6	24.3	20–24	
17	24	40.8	41.4	21–25	17–29
18	25	53.3	52.4	23–26	19–30
19	26	66.4	62.8	24–27	20–30
20	27	80.3	74.3	25–28	22–30
21	29	91.5	94.3	26–29	23–30
22	30	100	100	28–30	24–30

*Note*: MoCA‐22 scores ≤5 were extremely sparsely so conversion estimates rely on extrapolation of the log‐linear smoothed equating function rather than direct empirical support.

^a^ 95% prediction intervals represent the range within which 95% of individual observed MoCA‐30 scores are expected to fall for a given MoCA‐22.

^b^ 95% prediction intervals represent the range within which 95% of individual observed MoCA‐30 scores are expected to fall for a given T‐MoCA score, incorporating uncertainty in both the T‐MoCA to MoCA‐22 and MoCA‐22 to MoCA‐30 conversions. Intervals are reported only for scores with > 10 contributing observations to avoid estimation from sparsely represented score levels.

**TABLE 4:** Moca‐22 to MoCA‐30 conversion performance metrics across cohorts

Cohort	Method	*ρ*	Lin's CCC	RMSE	MAE	KS	CvM	W_2_
Development set								
OPDC discovery	Equating	0.95	0.95	1.16	0.85	0.060	0.012	0.59
OPDC discovery	Scaling	0.95	0.95	1.30	0.97	0.094	0.027	0.77
Validation sets								
Tracking PD	Equating	0.96	0.95	1.13	0.78	0.092	0.023	0.66
Tracking PD	Scaling	0.96	0.94	1.33	0.94	0.094	0.039	0.94
PPMI	Equating	0.93	0.94	1.04	0.71	0.085	0.019	0.59
PPMI	Scaling	0.93	0.93	1.13	0.77	0.085	0.023	0.69

Abbreviations: *ρ*, Spearman's *ρ*; CCC, Concordance Correlation Coefficient; KS, Kolmogorov–Smirnov statistic; CvM, Cramér–von Mises; W_2_, Wasserstein.

**Figure 2:**
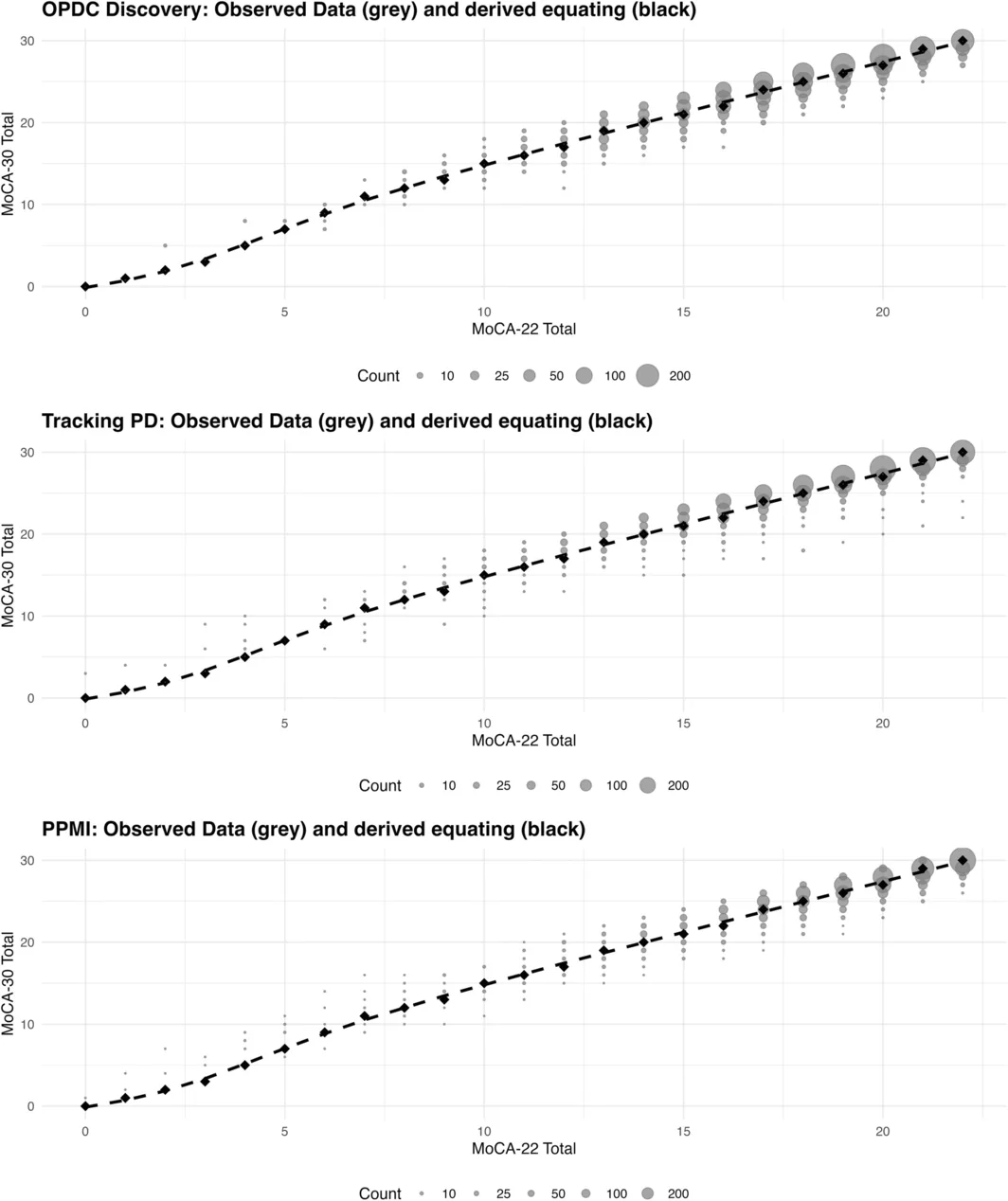
Scatterplots of paired MoCA‐22 and observed MoCA‐30 scores with the derived equating function applied in the three cohorts.

**Figure 3:**
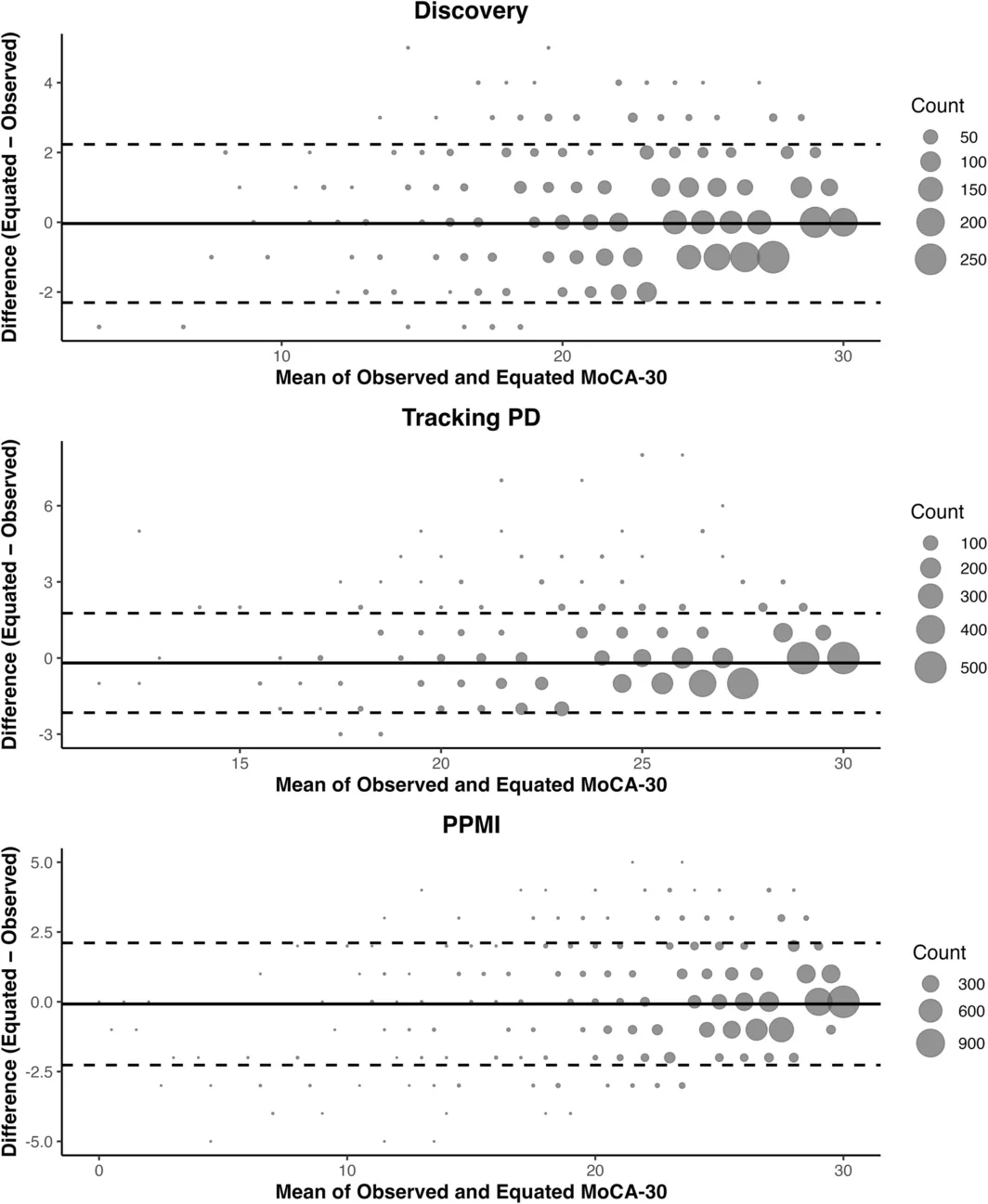
Bland–Altman plots of observed MoCA‐30 and equated MoCA‐30 scores with in the three cohorts.

### Validating the Equating Function

The derived equating function was subsequently applied to the MoCA‐22 data from two external datasets (Tracking Parkinson's and PPMI), and estimated MoCA‐30 scores were compared to observed MoCA‐30 scores within each cohort. Performance was evaluated using the same performance and distributional fit metric and results were compared with the reference method of simple proportional scaling. In the Tracking Parkinson's cohort, the equating method produced a Spearman's *ρ* of 0.96, a Lin's CCC of 0.95, an RMSE of 1.13 and an MAE of 0.78. The Kolmogorov–Smirnov statistic was 0.092, the Cramér–von Mises statistic 0.023, and the Wasserstein distance 0.66. Proportional scaling produced a Spearman's *ρ* of 0.96, a Lin's CCC of 0.94, an RMSE of 1.33, a MAE of 0.94, a Kolmogorov–Smirnov statistic of 0.094, a Cramér–von Mises statistic of 0.039, and a Wasserstein distance of 0.94 (Table 4). In the PPMI cohort, the equating method produced a Spearman's *ρ* of 0.93, a Lin's CCC of 0.94, an RMSE of 1.04 and an MAE of 0.71. The Kolmogorov–Smirnov statistic was 0.085, the Cramér–von Mises statistic 0.019, and the Wasserstein distance 0.59. By comparison, proportional scaling produced a Spearman's *ρ* of 0.93, a Lin's CCC of 0.93, an RMSE of 1.13, an MAE of 0.77, a Kolmogorov–Smirnov statistic of 0.085, a Cramér–von Mises statistic of 0.023 and a Wasserstein distance of 0.69 (Table 4). To address the non‐independent nature of repeated MoCA observations in the original dataset, a sensitivity analysis using bootstrap resampling was performed to repeatedly refit the equating function in the OPDC Discovery cohort and validate it in the Tracking Parkinson's and PPMI cohorts. The bootstrap results indicated that the equating function was robust, with close agreement between bootstrap estimates and the original values (Supplementary Fig. 4). Taken all together, the equating function's performance was comparable between the OPDC Discovery cohort (the development set) and the two external validation cohorts. Individual‐level prediction error was similar or slightly lower in the validation cohorts, while distributional agreement metrics were broadly consistent, with only modest increases in Kolmogorov–Smirnov statistics indicating slightly greater local discrepancies. For each respective cohort, the relationship between observed MoCA‐22 and MoCA‐30 scores and the derived equating function is illustrated in Figure 2 and agreement between observed and equated MoCA‐30 scores is shown in Figure 3.

To assess the potential clinical impact of using equated MoCA‐30 scores, we compared cognitive classification based on observed and equated MoCA‐30 scores, defining dementia as ≤20, mild cognitive impairment (MCI) as 21–25, and normal cognition as ≥26. Classification was unchanged in 86.6% of assessments in the OPDC Discovery cohort (Cohen's *κ* = 0.777), 88.2% in Tracking Parkinson's (Cohen's *κ* = 0.788), and 88.4% in PPMI (Cohen's *κ* = 0.738). Classification changes occurred almost exclusively between adjacent categories: dementia to MCI (OPDC Discovery: 2.0%; Tracking Parkinson's: 1.2%; PPMI: 1.0%), MCI to normal cognition (4.0%; 2.0%; 3.7%, respectively), MCI to dementia (2.5%; 2.6%; 1.5%, respectively), and normal cognition to MCI (4.9%; 5.9%; 5.4%, respectively). See Supplementary Tables 9 and 10 for further detail.

## Discussion

In this study, we evaluated the validity of the T‐MoCA in participants with iRBD and PD and derived a conversion method to harmonize 22‐point MoCA scores with standard 30‐point MoCA scores. When the T‐MoCA was compared with the face‐to‐face (F2F) MoCA‐22, T‐MoCA scores were statistically equivalent to F2F MoCA‐22 scores within the prespecified ±1‐point equivalence bounds. Median absolute differences were small and Bland–Altman analyses showed mean differences close to zero, indicating no systematic inflation or deflation of scores with telephone administration relative to F2F assessment. Although T‐MoCA and F2F MoCA‐22 scores were equivalent at the group level, performance was influenced by participant and testing factors. Males had modestly lower T‐MoCA scores than females, although this sex difference did not vary significantly by diagnostic group and no significant sex effect was observed for MoCA‐22. Test order also influenced performance, with scores tending to be lower on the second assessment, consistent with a possible fatigue effect. These findings suggest that sex and test order should be considered when interpreting T‐MoCA performance, although neither detracts from the overall group‐level equivalence between T‐MoCA and F2F MoCA‐22 observed in this study. Overall concordance and agreement between the two tests were moderate, which is not unexpected given the restricted score range of the 22‐point scale, the relatively high cognitive performance of many participants and the published estimates of MoCA test–retest reliability.^24^ Ceiling effects may also reduce between‐subject variability and attenuate correlation‐based metrics even when absolute differences between test formats are small.^25^ This distinction is important: the equivalence and Bland–Altman results indicate that the two formats yield similar scores at the group level, whereas the moderate CCC reflects variability at the individual level. The second objective of this study was to support comparability between non‐visual MoCA scores (MoCA‐22) and standard MoCA‐30 scores. In the OPDC Discovery cohort, equipercentile equating outperformed simple proportional scaling across both performance and distributional metrics. This performance was preserved when the same equating function was applied externally to PPMI and Tracking Parkinson's. Across all three cohorts, both conversion methods showed strong correlation and concordance with observed scores, but the equating function broadly produced lower prediction error and distributional fit metrics than proportional scaling. Although these differences were modest, they suggest that equipercentile equating better preserved both individual score agreement and overall score distribution. Equating also largely preserved cognitive classification, with observed and equated classifications agreeing in 86.6–89.3% of assessments across cohorts.

Taken together, these findings provide an evidence‐based rationale for using the T‐MoCA in cohort studies for cognitive follow‐up when F2F assessment is not feasible. Furthermore, in the absence of large‐scale PD cohorts with sufficient T‐MoCA data to derive and robustly validate a direct T‐MoCA‐to‐MoCA‐30 conversion, our validated MoCA‐22‐to‐MoCA‐30 conversion provides a pragmatic alternative, allowing telephone‐obtained 22‐point scores to be estimated on the more familiar 30‐point MoCA scale. The external validation strengthens this approach: although the three cohorts differed in their MoCA score distributions, the OPDC Discovery‐derived conversion function performed consistently in both external datasets, supporting generalizability of the conversion table across PD cohorts. Compared with the equipercentile T‐MoCA‐to‐MoCA‐30 conversion derived from a diverse non‐PD cohort (Katz et al.), the OPDC Discovery MoCA‐22 conversion produced slightly higher estimated MoCA‐30 scores across most of the scale, typically by 1 point.^8^ Previous studies in PD and non‐PD cohorts have suggested that a T‐MoCA or face‐to‐face MoCA‐22 score of ≤18/22 may be a reasonable threshold for MCI.^8^, ^10^, ^26^ Our conversion table translates a MoCA‐22 score of 18 to a MoCA‐30 score of 25, aligning the implied MCI threshold with that reported in the original MoCA‐30 validation study, where scores below 26 indicate potential cognitive impairment.^3^ However, a prudent consideration is the application of these converted scores at the individual level. Although the equating procedure performed well for harmonizing MoCA‐22 and MoCA‐30 scores at the cohort level, and individual‐level uncertainty associated with the MoCA‐22‐to‐MoCA‐30 conversion was relatively modest, additional uncertainty was introduced when the starting score was obtained by telephone. Propagating the observed variability between T‐MoCA and F2F MoCA‐22 through the subsequent MoCA‐22‐to‐MoCA‐30 conversion resulted in substantially wider prediction intervals for T‐MoCA‐derived MoCA‐30 estimates. This demonstrates that good cohort‐level equating performance does not imply equivalent precision for an individual MoCA‐30 estimate derived from T‐MoCA. Consequently, although the T‐MoCA, together with the MoCA‐22‐to‐MoCA‐30 conversion table, provides a pragmatic alternative when F2F assessment is not feasible, MoCA‐30 scores estimated from T‐MoCA should not be considered to have the same individual‐level precision as directly observed MoCA‐30 scores, and caution is required when using such estimates in relation to clinical thresholds for cognitive impairment.

Several limitations should be considered. First, the sample size of the T‐MoCA sub‐study was modest, particularly within diagnostic subgroups, and further validation in larger samples is needed. Second, participants in the T‐MoCA sub‐study generally had high MoCA scores, which may reflect recruitment‐related selection bias. Although most participants entered OPDC Discovery within 3 years of diagnosis, they were recruited for ton a rolling basis from those attending their scheduled study visits. Consequently, the mean disease duration at the time of the sub‐study was 11 years, and participants remaining in active follow‐up and able to complete the in‐person assessments likely represent a relatively cognitively preserved subgroup. This resulted in limited representation of individuals with moderate or severe cognitive impairment. The findings may therefore be less generalizable to individuals with more advanced cognitive impairment or PD dementia. Third, the T‐MoCA and F2F MoCA‐22 were administered within a relatively short time interval and as part of a broader research visit, and although test order was balanced across groups, fatigue could have influenced agreement between formats. Fourth, the T‐MoCA omits visuospatial/executive and naming items by design. These domains may be clinically informative in PD, and telephone assessment cannot fully substitute for a F2F MoCA‐30 where visuospatial deficits are of particular relevance.^27^ Excluding visual items may therefore underestimate cognitive impairment in PD. However, our findings suggest the impact may be modest at the group level, as observed/equated cognitive classification agreed in approximately 90% of assessments across all three cohorts, with Cohen's *κ* indicating substantial agreement. Fifth, visual and hearing impairment were not formally assessed or controlled for, and their potential influence on test performance cannot be excluded.

In summary, this study demonstrated that the T‐MoCA was statistically equivalent to F2F MoCA‐22 at the group level, although moderate individual‐level concordance supports cautious interpretation of individual scores. In addition, equipercentile equating provided a robust and externally validated method for converting MoCA‐22 scores to estimated MoCA‐30 scores, both in PD and iRBD. Together, these findings support the use of the T‐MoCA as a cognitive screening tool when face‐to‐face assessment is not feasible and provide a conversion framework for harmonizing remote and in‐person MoCA data in PD and prodromal PD research.

## Author Roles

(1) Research project: A. Conception, B. Organization, C. Execution; (2) Statistical Analysis: A. Design, B. Execution, C. Review and Critique; (3) Manuscript Preparation: A. Writing of the first draft, B. Review and Critique.

L.v.H.: 1A, 1B, 1C, 2A, 2B, 3A.

T.Z.: 2A, 2B, 2C, 3B.

M.L.: 2C, 3B.

K.G.: 1B, 1C, 3B.

J.R.: 1B, 1C, 3B.

J.W.: 1B, 1C, 3B.

Y.B‐S.: 2C, 3B.

M.H.: 1A, 1B, 2C, 3B.

## Disclosures

**Ethical Compliance Statement:** The OPDC Discovery and PPMI cohort studies were conducted in accordance with the Declaration of Helsinki and Good Clinical Practice guidelines and was approved by the local ethics committees of the participating sites. All participants provided written informed consent. We confirm that we have read the Journal's position on issues involved in ethical publication and affirm that this work is consistent with those guidelines.

**Funding Sources and Conflicts of Interest:** This work was funded by a grant from Parkinson's UK. M.H. is an advisory founder, paid consultant and shareholder of KneuHealth Digital Ltd, a digital health company with interests in remote motor and cognitive assessment. This relationship was not involved in the design, conduct, analysis, or reporting of this study. The authors declare no other conflicts of interest relevant to this work.

**Financial Disclosures and Conflicts of Interest:** L.v.H., K.G. and J.W. are employed by the University of Oxford. J.R is employed by Oxford University Hospitals NHS Foundation Trust. M.L. and Y.B‐S are employed by the University of Bristol. T.Z. received funding/grant support from Parkinson's UK. Y.BS received consultancy payment from Parkinson's UK for an unrelated study on the epidemiology of PD. M.H. received funding/grant support from Parkinson's UK, Oxford NIHR BRC, University of Oxford, CPT, Lab10X, NIHR, Michael J Fox Foundation, European Platform for Neurodegenerative Disorders) GE Healthcare and the PSP Association. M.H. has received payment for Advisory Board attendance/consultancy from Helicon, NeuHealth Digital, Roche and Manus Neurodynamica. M.H. is an advisory founder, paid consultant and shareholder of KneuHealth Digital Ltd (company number: 14492037), and joint patent holder (18/815,353) to predict dopamine neuron loss in Parkinson's based on simulated dopamine transporter scan (DaTScan) tracer uptake using sensor and clinical data. Author disclosures are available in the Supporting Information.

## Supporting information


**Supplementary Figure 1.** Density plots of MoCA test scores in the three PD cohorts.
**Supplementary Table 1.** Regression analyses testing effect of test format and order.
**Supplementary Table 2.** Linear regression analyses examining associations of sex and diagnostic group with T‐MoCA and MoCA‐22 scores.
**Supplementary Table 3.** Effect of education on the relationship between MoCA‐22 and MoCA‐30 scores.
**Supplementary Figure 2.** Distribution of errors across MoCA items.
**Supplementary Table 4.** Points lost on visual and non‐visual Montreal cognitive assessment items across cohorts.
**Supplementary Table 5.** Comparison of equipercentile conversion table derived from the PD and iRBD arms of the OPDC discovery cohort.
**Supplementary Table 6.** Performance of MoCA‐22‐to‐MoCA‐30 equating functions derived separately in the PD and iRBD arms of the OPDC Discovery cohort.
**Supplementary Table 7.** Equating residual of the PD‐derived MoCA‐22‐to‐MoCA‐30 equating function in the PD and iRBD arms of the OPDC Discovery cohort.
**Supplementary Table 8.** Linear mixed‐effects model of equating residual by disease duration in the PD arm of the OPDC Discovery cohort.
**Supplementary Figure 3.** Equating residual and observed MoCA‐30 scores across disease duration in the OPDC Discovery cohort (PD arm).
**Supplementary Figure 4.** Bootstrap validation of OPDC Discovery‐derived equipercentile equating formula.
**Supplementary Table 9.** Agreement between observed and equated MoCA‐30 cognitive classifications.
**Supplementary Table 10.** Classification agreement between observed and equated MoCA‐30.

**Data S1.** Disclosure Statement form.

## Data Availability

The data that support the findings of this study are available on request from the corresponding author. The data are not publicly available due to privacy or ethical restrictions.
