# Supplementary material for: Validation of the Telephone MoCA in iRBD and Parkinson's Disease with Conversion to the Standard MoCA
Source: Mov Disord Clin Pract. 2026 Oct 30:10.1002/mdc3.70905. Online ahead of print. doi: 10.1002/mdc3.70905 (PMC13722836; doi:10.1002/mdc3.70905)
Supplement: Supplementary file 2 [file MDC3-9999-0-s002.docx]

**SUPPLEMENTARY MATERIAL**

**Supplementary Figure 1.** Density plots of MoCA test scores in the three PD cohorts

**Supplementary Table 1.** Regression analyses testing effect of test format and order

|  | *Linear mixed effects model: score ~ format * order + (1 \| subject ID)* | | | | | |  |
| --- | --- | --- | --- | --- | --- | --- | --- |
|  |  |  |  | ***β*** | **SE** | ***p*-value** |  |
|  | Intercept |  |  | 19.30 | 0.27 | 2×10^-16^ |  |
|  | Format: T-MoCA |  |  | -0.58 | 0.24 | 0.015 |  |
|  | Order: T-MoCA first |  |  | -0.58 | 0.40 | 0.14 |  |
|  | Format x Order |  |  | 1.43 | 0.34 | 2×10^-16^ |  |
|  |  |  |  |  |  |  |  |
|  | *Estimated format differences within each order group* | | | | | |  |
|  |  |  | **Contrast** | ***β*** | **SE** | ***p*-value** |  |
|  | MoCA-22 first |  | T-MoCA – F2F MoCA-22 | -0.58 | 0.24 | 0.015 |  |
|  | T-MOCA first |  | T-MoCA – F2F MoCA-22 | 0.85 | 0.25 | 0.0009 |  |

*T-MoCA = Telephone Montreal Cognitive Assessment. β represents the estimated regression coefficient; SE = standard error.*

**Supplementary Table 2.** Linear regression analyses examining associations of sex and diagnostic group with T-MoCA and MoCA-22 scores

| Outcome / Predictor | *β* | SE | t | *p*-value |
| --- | --- | --- | --- | --- |
| *T-MoCA: overall sex model* |  |  |  |  |
| Male sex | -0.99 | 0.41 | -2.43 | 0.016 |
| *T-MoCA: sex × diagnosis model* |  |  |  |  |
| Male sex | -1.20 | 0.73 | -1.64 | 0.104 |
| iRBD | 0.80 | 1.40 | 0.57 | 0.568 |
| Parkinson’s disease | -0.96 | 0.68 | -1.42 | 0.159 |
| Male × iRBD | -0.87 | 1.53 | -0.57 | 0.573 |
| Male × Parkinson’s disease | 0.29 | 0.97 | 0.30 | 0.762 |
| *MoCA-22 — overall sex model* |  |  |  |  |
| Male sex | -0.68 | 0.42 | -1.62 | 0.108 |
| *MoCA-22 — sex × diagnosis model* |  |  |  |  |
| Male sex | -1.04 | 0.77 | -1.35 | 0.179 |
| iRBD | -1.15 | 1.46 | -0.79 | 0.434 |
| Parkinson’s disease | -1.19 | 0.71 | -1.68 | 0.096 |
| Male × iRBD | 0.70 | 1.61 | 0.43 | 0.665 |
| Male × Parkinson’s disease | 0.82 | 1.01 | 0.81 | 0.419 |

*Female sex and healthy controls are the reference categories. T-MoCA = Telephone Montreal Cognitive Assessment. β = regression coefficient; SE = standard error; iRBD = isolated REM sleep behaviour disorder. Note: the iRBD sex comparison is based on 3 females versus 44 males. A non-significant result should therefore not be interpreted as demonstrating the absence of a sex effect.*

**Supplementary Table 3.** Effect of education on the relationship between MoCA-22 and MoCA-30 scores

| Education measure | Cohort | β | SE | df | *p*-value |
| --- | --- | --- | --- | --- | --- |
| *Continuous education (years)* | OPDC Discovery | −0.0035 | 0.0019 | 2,863 | 0.070 |
|  | PPMI | 0.0086 | 0.0021 | 4,174 | 5.18x10^-05^ |
|  |  |  |  |  |  |
| *Binary education (≤12 vs >12 years)* | OPDC Discovery | 0.0151 | 0.0145 | 2,964 | 0.300 |
|  | Tracking Parkinson’s | 0.0419 | 0.0107 | 4,533 | 8.48x10^-05^ |
|  | PPMI | −0.0368 | 0.0177 | 4,324 | 0.038 |

*β represents the MoCA-22 × education interaction coefficient from linear mixed-effects models with participant-specific random intercepts to account for repeated assessments. MoCA-22 was mean-centred. For continuous education, β represents the change in the MoCA-22–MoCA-30 slope associated with each additional year of education. For binary education, education was coded as >12 years = 0 and ≤12 years = 1, therefore β represents the difference in the MoCA-22–MoCA-30 slope for participants with ≤12 years compared with those with >12 years of education.*

**Supplementary Figure 2.** Distribution of errors across MoCA items

**Supplementary Table 4.** Points lost on visual and non-visual Montreal Cognitive Assessment items across cohorts

|  | **OPDC Discovery** | **Tracking PD** | **PPMI** |
| --- | --- | --- | --- |
| Visual test items | 1 (0,2) | 0 (0,1) | 0 (0,1) |
| Non-visual test items | 4 (2,6) | 3 (1,5) | 2 (1,4) |

*Continuous values expressed as median (Q1, Q3).*

*Visual test items: Trail Making, Cube Copy, Clock Draw, Naming*

*Non-visual test items: Digit Span, Vigilance, Serial 7’s, Sentence Repetition, Phonemic Fluency, Abstraction, Delayed Recall, Orientation.*

**Supplementary Table 5.** Comparison of equipercentile conversion table derived from the PD and iRBD arms of the OPDC Discovery cohort

| MoCA-22 | Equated MoCA-30  derived from PD arm | Equated MoCA-30 derived from iRBD arm | Difference |
| --- | --- | --- | --- |
| 0 | 0 | 0 | 0 |
| 1 | 1 | 1 | 0 |
| 2 | 2 | 3 | 1 |
| 3 | 3 | 6 | 3 |
| 4 | 5 | 8 | 3 |
| 5 | 7 | 10 | 3 |
| 6 | 9 | 11 | 2 |
| 7 | 11 | 13 | 2 |
| 8 | 12 | 14 | 2 |
| 9 | 13 | 15 | 2 |
| 10 | 15 | 16 | 1 |
| 11 | 16 | 17 | 1 |
| 12 | 17 | 18 | 1 |
| 13 | 19 | 19 | 0 |
| 14 | 20 | 20 | 0 |
| 15 | 21 | 22 | 1 |
| 16 | 22 | 23 | 1 |
| 17 | 24 | 24 | 0 |
| 18 | 25 | 25 | 0 |
| 19 | 26 | 26 | 0 |
| 20 | 27 | 27 | 0 |
| 21 | 29 | 29 | 0 |
| 22 | 30 | 30 | 0 |

**Supplementary Table 6.** Performance of MoCA-22-to-MoCA-30 equating functions derived separately in the PD and iRBD arms of the OPDC Discovery cohort.

|  | **Study arm** |  | **Method** | **RMSE** | **MAE** | **KS** | **CvM** | **W_2_** |  |
| --- | --- | --- | --- | --- | --- | --- | --- | --- | --- |
|  | *PD* |  | Equating | 1.16 | 0.85 | 0.060 | 0.012 | 0.59 |  |
|  | *iRBD* |  | Equating | 1.06 | 0.77 | 0.074 | 0.012 | 0.56 |  |

*PD = Parkinson’s disease. iRBD = isolated REM-sleep behaviour disorder*

*KS = Kolmogorov–Smirnov statistic. CvM = Cramér–von Mises. W_2_ = Wasserstein*

**Supplementary Table 7.** Equating residual of the PD-derived MoCA-22-to-MoCA-30 equating function in the PD and iRBD arms of the OPDC Discovery cohort

**A)** Prediction-error metrics

| Study arm | Mean residual (SD) | Median residual (IQR) | MAE | RMSE |
| --- | --- | --- | --- | --- |
| PD | -0.04 (1.16) | 0 (2) | 0.85 | 1.16 |
| iRBD | -0.22 (1.11) | 0 (1) | 0.85 | 1.13 |

**B)** Linear mixed-effects model of equating residual by study arm

Formula: *Equating residual_ij_ = β_0_ + β_1_(iRBD_i_) + u_i_ + ε_ij_*

| Fixed effect | β | SE | df | t | *p*-value |
| --- | --- | --- | --- | --- | --- |
| Intercept | -0.017 | 0.026 | 1,069 | 0.68 | 0.497 |
| iRBD arm vs PD arm | -0.192 | 0.053 | 1,088 | 3.60 | 0.0003 |

*MAE = mean absolute error; RMSE = root mean squared error; PD = Parkinson’s disease; iRBD = isolated REM sleep behaviour disorder. β represents the estimated regression coefficient; SE = standard error; df = degrees of freedom. Equating residual was defined as equated MoCA-30 using the PD-derived equating function minus observed MoCA-30. The linear mixed-effects model included study arm as a fixed effect and participant as a random intercept to account for repeated assessments (4,630 assessments from 1,313 participants).*

**Supplementary Table 8.** Linear mixed-effects model of equating residual by disease duration in the PD arm of the OPDC Discovery cohort

Formula: *Equating residual_ij_ = β_0_ + β_1_(age) + β_2_(sex) + β_3_(disease duration) + u_i_ + ε_ij_*

| Fixed effect | β | SE | df | t | *p*-value |
| --- | --- | --- | --- | --- | --- |
| Intercept | −0.817 | 0.237 | 901 | −3.44 | 0.0006 |
| Age (years) | 0.013 | 0.003 | 885 | 4.62 | 4.32x10^-06^ |
| Sex (ref. female) | -0.099 | 0.054 | 807 | -1.86 | 0.063 |
| Education (years) | −0.011 | 0.007 | 795 | −1.47 | 0.142 |
| Disease duration (years) | 0.011 | 0.006 | 3,406 | 1.84 | 0.066 |

*Equating residual was defined as equated MoCA-30 using the PD-derived equating function minus observed MoCA-30. The linear mixed-effects model included age, sex, education and disease duration as fixed effects and a participant-specific random intercept to account for repeated longitudinal assessments (3,547 assessments from 1,003 participants). β represents the estimated regression coefficient; SE = standard error; df = degrees of freedom.*

**Supplementary Figure 3. Equating residual and observed MoCA-30 scores across disease duration in the OPDC Discovery cohort (PD arm).**

*Assessments were grouped into 2-year disease-duration intervals. Points and solid lines represent the mean equating residual (equated minus observed MoCA-30), with error bars indicating 95% confidence intervals. The dotted line with triangular markers represents the mean observed MoCA-30 score within each interval, plotted against the secondary y-axis.*

n = *the number of MoCA assessments contributing to each disease-duration interval.*

**Supplementary Figure 4.** Bootstrap validation of OPDC Discovery-derived equipercentile equating formula

*Bootstrap resampling (500 iterations) was used to repeatedly draw independent MoCA scores (one per participant) from the OPDC Discovery cohort, refit the equipercentile equating function, and validate the formula in the Tracking PD and PPMI. Points denote bootstrap means and whiskers denote the 2.5th–97.5th percentile interval. RMSE = Root mean square error. MAE = Mean absolute error*

**X** = *RMSE and MAE from original OPDC Discovery development set which includes longitudinal MoCA scores*

**Supplementary Table 9.** Agreement between observed and equated MoCA-30 cognitive classifications

| Cohort | n | Agreement (%) | Cohen’s κ |
| --- | --- | --- | --- |
| *Discovery* | 3,561 | 86.6 | 0.78 |
| *Tracking PD* | 5,767 | 88.2 | 0.79 |
| *PPMI* | 5,047 | 88.2 | 0.79 |

*Agreement represents the percentage of assessments assigned to the same cognitive category using the observed and equated MoCA-30 scores. Cognitive categories were defined as dementia (<21), mild cognitive impairment (MCI; 21-25), and normal (≥26). Cohen’s κ represents agreement after accounting for agreement expected by chance.*

**Supplementary Table 10.** Classification agreement between observed and equated MoCA-30

| **Classification outcome** | **Discovery** | **Tracking PD** | **PPMI** |
| --- | --- | --- | --- |
| No change | 3,083 (86.6) | 5,085 (88.2) | 4,509 (89.3) |
| **Equated classification higher** |  |  |  |
| Dementia → MCI | 72 (2.0) | 72 (1.2) | 41 (0.8) |
| MCI → Normal | 142 (4.0) | 117 (2.0) | 168 (3.3) |
| Dementia → Normal | 0 (0.0) | 2 (<0.1) | 0 (0.0) |
| **Equated classification lower** |  |  |  |
| Normal → MCI | 176 (4.9) | 340 (5.9) | 272 (5.4) |
| MCI → Dementia | 88 (2.5) | 151 (2.6) | 57 (1.1) |
| Normal → Dementia | 0 (0.0) | 0 (0.0) | 0 (0.0) |
| **Total** | 3,561 (100) | 5,767 (100) | 5,047 (100) |

*Values expressed as n, (%). Cognitive categories were defined as dementia (<21), mild cognitive impairment (MCI; 21-25), and normal (≥26). “Equated classification higher” indicates that the equated MoCA-30 placed the assessment in a less impaired category than the observed MoCA-30; “Equated classification lower” indicates classification into a more impaired category.*
